# Clinical Management of *Helicobacter* Infection in Captive Baboons ( *Papio anubis* and *Papio hamadryas* ), and Isolation and Molecular Characterization of Three Novel *Helicobacter* Species

**DOI:** 10.1111/jmp.70095

**Published:** 2026-06-19

**Authors:** Isabel A. Jimenez, Courtney Lunger, Marcus J. Crim, Shannon L. Primm, Anthony Mannion, Nathan P. Crilly, Lydia M. Hopper, Lindsey G. Thurman, Linnette L. Vasquez, Cory Brayton, Yan Feng, James G. Fox, Zeli Shen, Jessica M. Izzi

**Affiliations:** ^1^ Department of Molecular and Comparative Pathobiology Johns Hopkins University School of Medicine Baltimore Maryland USA; ^2^ Division of Comparative Medicine Massachusetts Institute of Technology Cambridge Massachusetts USA; ^3^ IDEXX BioAnalytics Columbia Missouri USA; ^4^ Research Animal Resources Johns Hopkins University Baltimore Maryland USA

**Keywords:** captive management, enterohepatic *Helicobacter*, fecal PCR, surveillance

## Abstract

**Background:**

*Helicobacter* spp. are gastric or enterohepatic bacteria that can be commensal, opportunistic, or pathogenic. Few studies have evaluated *Helicobacter* spp. in baboons.

**Methods:**

Twenty‐seven captive baboons were surveyed for *Helicobacter* spp. by fecal PCR, and a subset evaluated by culture. Nine baboons were treated with oral “triple therapy” with clarithromycin (10 mg/kg), amoxicillin (10 mg/kg), and omeprazole (20 mg) once daily for 10 days.

**Results:**

All baboons were positive for *Helicobacter* spp., including *H. macacae* (78%), *H. suis* (19%), and *H. pylori* (15%). Three novel *Helicobacter* species were identified by whole genomic sequencing, for which we propose the names *Helicobacter papionis*, *H. simiae*, and *H. papionifaecis.* Treatment was safe and effective for *H. pylori* , *H. suis* , and *H. macacae*, but did not eliminate the novel *Helicobacter* spp.

**Conclusions:**

Species‐specific assays should guide treatment of *Helicobacter* spp. infections in baboons. Further epidemiologic studies are needed to inform clinical management.

**Abbreviations**
EHS: enterohepatic *Helicobacter* species
*gyrB*
: DNA gyrase B
HHLOs:
*H. heilmannii* ‐like organisms
*hsp60*
: heat shock protein 60
NHPs: non‐human primates
PCR: polymerase chain reaction
*rpoB*
: RNA polymerase subunit B

## 1 Introduction

The genus *Helicobacter* comprises Gram‐negative, spiral‐shaped, microaerophilic, fastidious bacteria grouped by tissue tropism: gastric species colonize the stomach while enterohepatic *Helicobacter* species (EHS) colonize the small intestine, large intestine, and hepatobiliary tree. Gastric species include *H. pylori* and *H. heilmannii* ‐like organisms (HHLOs) [1, 2]. *H. pylori* is a human pathogen linked to gastritis, gastric ulceration, gastric adenocarcinoma, gastric lymphoma, and rarely inflammatory bowel disease (IBD) [3, 4]. EHS are associated with IBD, enteritis, colitis, and proctitis, bacteremia, and neoplasia in humans, often in immunocompromised patients [5, 6, 7]. In humans, non‐human primates (NHPs), dogs, and cats, *Helicobacter* spp. infection may be subclinical, but during illness may also be opportunistic pathogens [8, 9, 10, 11].

Among NHPs, *Helicobacter* spp. have been most extensively studied in macaques, with gastric species including *H. pylori* , HHLOs, and *H. suis* , and EHS including *H. cinaedi* and *H. macacae* [12, 13, 14, 15]. Infection in macaques may be asymptomatic or associated with gastritis, chronic diarrhea, and intestinal adenocarcinoma [12, 16, 17, 18, 19]. There is limited literature regarding *Helicobacter* in baboons (Table S1), with gastric species more often described. *H. pylori* and HHLOs have been observed in the gastric mucosa and parietal cells of wild‐born olive baboons ( *Papio anubis* ) [20, 21] but are not always present, even in baboons with lymphoplasmacytic gastritis [16, 22]. While one group posited HHLOs were commensal, others found HHLOs to be associated with gastritis [23, 24]. Two studies have isolated EHS from baboons, with homology to *H. macacae* and *H. cinaedi* but unknown clinical relevance [25, 26]. It is unclear if *Helicobacter* are commensals, pathobionts, or pathogens in baboons.

Here, a case of gastritis in an olive baboon led to detection of an unidentified *Helicobacter* spp. and its genetic sequence, prompting colony‐wide surveillance to assess the prevalence of *Helicobacter* spp. in our baboons and ultimately leading to the isolation and characterization of three novel *Helicobacter* species.

## 2 Materials and Methods

### 2.1 Study Population and Humane Care Guidelines

Twenty‐seven baboons from two colonies, including 11 male and 1 female olive baboons ( *Papio anubis* ) from colony A, and 14 male olive baboons and 1 male hamadryas baboon ( *Papio hamadryas* ) from colony B, were included (Table S2). All baboons in this study were housed at Johns Hopkins University (JHU) at an AAALAC International‐accredited facility, in accordance with the Animal Welfare Act (7 U.S.C. 2131), Animal Welfare Regulations (Title 9 C.F.R.) and the 8th Edition of the *Guide for the Care and Use of Laboratory Animals* [27]. Baboons were maintained under protocols approved by the JHU Animal Care and Use Committee and were singly‐housed for protocol‐related purposes or behavioral management. The authors confirm that the ethical policies of the *Journal of Medical Primatology*, as noted on the journal's author guidelines page, have been followed. Animals in this study were not on protocols investigating hepatobiliary or gastrointestinal (GI) disease, or infectious disease. Colonies were 4 miles apart, in buildings that also housed rodents; the facility with Colony A also housed macaques, marmosets, pigs, sheep, dogs, cats, and ferrets.

### 2.2 Data Collection

Medical records were reviewed for age, origin (captive‐bred vs. wild‐born, facility of birth, intermediate facility, facility immediately prior to arrival at our institution) and tenure at our facility (Table S2). Four baboons were originally caught from the wild (all *Papio anubis* ), and the remaining animals were captive‐bred. Baboons arrived from six facilities. Ages ranged from 3.5 to 27.3 years (mean 18.3 years) with a tenure of 2–21.9 years (mean 13.7 years). The date of birth for wild‐born animals was estimated as 1 year prior to capture or the earliest medical record. Medical records were examined for GI signs such as inappetence, vomiting, regurgitation, diarrhea, constipation, or hematochezia. Eight baboons had a history of acute GI signs, all either self‐limiting or resolved with treatment before the start of study (Table S2). No major medical history was present in any animals. Major medical history was defined as clinical concerns (apart from superficial wounds, dermal masses, dental pathology, osteoarthritis, or alopecia) or sedation/anesthetic events (apart from routine preventive or protocol‐related procedures). No animals had prior fecal PCR testing for *Helicobacter* spp. or were previously treated for *Helicobacter* spp.

### 2.3 Sample Collection

For PCR, feces were collected and stored at 4°C for up to 24 h or frozen at −80°C until shipment to a diagnostic laboratory (IDEXX BioAnalytics, Columbia, MO 65201). In Colony A, additional fecal samples were collected 2 weeks and 14–16 months after completing antibiotic treatment. For culture, fecal samples from 16 baboons (seven from Colony A collected at least 18 months after completion of antibiotics, and 9 from Colony B) were stored in 20% glycerol in Brucella broth at −80°C until shipment on dry ice to MIT. In a subset of animals euthanized for unrelated reasons, post‐mortem fecal, gastric, gall bladder, liver, small intestine, and colon samples were collected and stored at −20°C for up to 24 h, then frozen at −80°C until shipment to the diagnostic laboratory.

### 2.4 Histopathology

Tissue samples were fixed in 10% neutral‐buffered formalin and paraffin‐embedded. Sections were stained with hematoxylin and eosin or Warthin–Starry and examined by a board‐certified veterinary anatomic pathologist. Images were collected on a Nikon Eclipse 80i microscope with a Y‐TV55 adaptor and Morrell camera and processed in Adobe Photoshop 25.9.0, following anatomic pathology guidelines [28].

### 2.5 Fecal Sample Real‐Time PCR, Conventional PCR, and Sanger Sequencing

Fecal samples underwent genus‐wide *Helicobacter* spp. real‐time PCR assay and species‐specific real‐time PCR assays to detect *H. cinaedi/canicola*, *H. macacae*, *H. pylori* , and *H. suis* . Real‐time PCR assays were based on the IDEXX BioAnalytics proprietary service platform (IDEXX Laboratories, Westbrook, ME). Samples were homogenized and total nucleic acids were extracted and tested by fluorogenic hydrolysis probe‐based real‐time PCR assays using a commercially available instrument (LightCycler 480, Roche Applied Science, Indianapolis, IN) and master mix (LightCycler 480 Probes Master, Roche Applied Science). Assay‐specific positive and negative controls were included for each run and positive samples were retested for confirmation. A multiplexed hydrolysis‐probe‐based real‐time PCR assay targeting a eukaryotic gene (*18SrRNA*) and bacterial gene (*16SrRNA*) was performed for each sample to demonstrate nucleic acid recovery and absence of PCR inhibition. To limit uninterpretable sequencing results due to coinfection, only samples from animals negative for the four species‐specific real‐time PCR assays but positive on genus‐wide *Helicobacter* spp. assay were assessed for atypical *Helicobacter* sequences. The literature suggests that *16SrRNA* sequences should not be used to solely identify unknown *Helicobacter* isolates, as they are often highly conserved among *Helicobacter* spp. [29, 30, 31] Moreover, there is some evidence for horizontal *16*S*rRNA* gene fragment transfers; thus, *16SrRNA* similarity does not always correctly reflect *Helicobacter* phylogenetic relationships [29, 32, 33]. We therefore also utilized additional genes throughout this study. The *16SrRNA*, *23SrRNA*, and/or *gyrB* genes were amplified using conventional PCR (primers shown in Table S3) then submitted for Sanger sequencing (GENEWIZ, South Plainfield, NJ) [34]. BioEdit [35] v.7.2.5 and Sequencher software (Gene Codes Corporation, Ann Arbor, MI) were used to assemble reads into contigs. Regions of poor‐quality sequence and primer binding sequences were removed. Sequencing results with multiple traces were excluded. Contigs were compared to GenBank sequences and to whole genome sequences of novel species subsequently identified in this study using BLAST software [36].

### 2.6 Antibiotic Treatment

Oral “triple therapy” of amoxicillin (10 mg/kg), clarithromycin (10 mg/kg), and omeprazole (20 mg/baboon) once per day for 10 days was given to nine baboons in colony A. Baboons did not receive other antibiotics between diagnosis and follow‐up fecal PCRs.

### 2.7 Bacterial Isolation

Feces in freeze media (~200 mg) were directly placed on CVA (cefoperazone, vancomycin, and amphotericin) or TVP (trimethoprim, vancomycin, and polymyxin B) plates (Remel Laboratories, Lenexa, KS), or filtered through 0.65 or 0.45 μm filters onto blood agar plates. Plates were incubated under microaerobic conditions (80% N_2_, 10% CO_2_, and 10% H_2_) at 37°C. Suspected *Helicobacter* spp. were identified by morphology and Gram stain.

### 2.8 Pure Isolate and Paraffin‐Embedded Tumor DNA Extraction, PCR and Sanger Sequencing

Microbial DNA was extracted from pure isolates using the Roche High Pure PCR Template Preparation Kit (Roche Life Science, Basel, Switzerland) according to manufacturer protocols. Isolates were confirmed as *Helicobacter* species by *16S rRNA* sequencing using conserved primers (Table S3). Isolates were speciated by heat shock protein 60 (*hsp60*) and RNA polymerase subunit B (*rpoB*) sequencing, which has been previously used to identify phylogenetic relationships among *Helicobacter* species [30, 33]; primers are shown in Table S3. PCR‐amplified products were subjected to Sanger sequencing. Sequence alignments and phylogenetic analysis were performed via Geneious bioinformatics software (Geneious v.7.1.7, Biomatters Ltd., Auckland, New Zealand). For *Helicobacter* PCR of paraffin‐embedded tumor, the Qiagen FFPE tissue kit (Qiagen, Hilden, Germany) was used to extract DNA. PCR was performed with C97/H676 and C97/C98N primers (Table S3) using the High‐Fidelity PCR System (Roche Diagnostics, Indianapolis, IN).

### 2.9 Whole Genome Sequencing

Genomic DNA was prepared for Illumina MiSeq sequencing (2 × 300 bp) by the BioMicroCenter at MIT using the Nextera Flex library prep kit. Default parameters were used for all bioinformatics tools. Raw sequenced reads were decontaminated of adapter sequences and quality trimmed using BBDuk (v.37.17; parameters: ktrimr, k23, mink11, hdist1, tpe, tbo, qtrimrl, trimq10, and qin33) [37] followed by de novo contig assembly and genome annotation using the Comprehensive Genome Analysis pipeline (The Bacterial and Viral Bioinformatics Resource Center (BV‐BRC), https://www.bv‐brc.org/). Genomes were deposited in NCBI under GenBank Accession numbers in Table S6. Using OrthoFinder [38], concatenated core protein sequences were determined, followed by MAFFT for multi‐sequence alignment and FastTree to infer approximate maximum‐likelihood phylogenetic trees. Taxonomic classification of genomes was supported by average nucleotide identity (pyANI, v.0.2.10) [39] and digital DNA–DNA hybridization (Genome‐to‐Genome Distance Calculator, v.3.0) [40]. Protein annotations were searched for the virulence factors flagellin, gamma‐glutamyltranspeptidase, High‐Temperature Requirement A, Cytotoxin‐associated gene A, Vacuolating cytotoxin A, Neutrophil‐activating protein A, catalase, Cytolethal distending toxin, Campylobacter invasion antigen B, and Fibronectin‐like protein A.

### 2.10 Biochemical Characterization

The RapID NH System (Remel Laboratories, Lenexa, KS) was performed, including tests for oxidase, catalase, nitrate reduction, indoxyl acetate hydrolysis, urease, phosphatase activity, γ‐glutamyl transferase, growth at 42°C, growth with 1% glycine, and resistance to nalidixic acid and cephalothin (30 mg disks) [41].

### 2.11 Transmission Electron Microscopy

Two isolates per novel species were imaged by transmission electron microscopy. 24–48‐h blood agar cultures were collected into 1× PBS. Bacterial concentration was adjusted to 0.05 OD/mL with PBS to decrease background effects. Samples were placed on formvar/carbon mesh grids (Electron Microscopy Sciences, Montgomery County, PA), negatively stained with Uranyless Solution (Electron Microscopy Sciences, Montgomery County, PA), and examined under a Jeol 2100F transmission electron microscope.

### 2.12 Statistical Analysis

Each animal was coded as 1 (positive) or 0 (negative) for *H. macacae*, *H. suis* , or *H. pylori* . A binomial general linear mixed model was used to compare infection rates by housing colony (A vs. B), housing room (A1 vs. A2 vs. B1 vs. B2), origin (wild‐born or captive‐born), birth location for captive‐born (one of three facilities), and housing facility prior to arrival at our institution (one of six facilities). A logistic regression was performed to assess the effect of age and tenure at our facility on the likelihood of being positive with *H. macacae*, *H. suis* , or *H. pylori* . Analyses were run in R v.4.1.1 [42].

## 3 Results

### 3.1 Case Study 1

During a routine experimental procedure, a 16‐year‐old intact male olive baboon (baboon #1, colony A) demonstrated marked regurgitation of partially digested blood. Gastric endoscopy revealed erythematous and inflamed mucosa without obvious ulceration. Three gastric mucosal biopsies were collected from separate sites. Radiographs revealed right cranial lung lobe consolidation, concerning for aspiration pneumonia. The patient was managed with gastroprotectants, antinausea medication, and antibiotics (enrofloxacin 5 mg/kg IM for 5 days). Gastric biopsy demonstrated frequent argyrophilic spiral organisms within gastric glands, consistent with *Helicobacter* spp., and mild neutrophilic inflammation (Figure 1). Fecal PCR was positive for *Helicobacter* spp., but negative for *H. macacae*, *H. pylori* , *H. suis* , and *H. cinaedi* . A portion of the *Helicobacter 16SrRNA* gene was sequenced from fecal nucleic acids, revealing an unidentified *Helicobacter* sequence.

**FIGURE 1:**
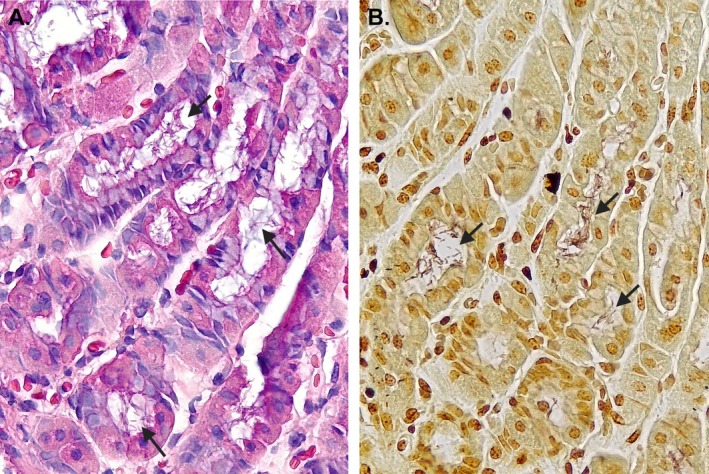
16‐year‐old male baboon ( *Papio anubis* ) with hematemesis; gastric mucosa (endoscopic biopsy). (A) Mild‐to‐moderate neutrophilic gastritis, abundant curved bacteria in gastric glands (arrows), mild submucosal fibrosis, H&E. (B) Argyrophilic curved bacteria, consistent with *Helicobacter* spp. (arrows), Warthin–Starry. Magnification: 40×.

### 3.2 Colony Survey

Twenty‐seven baboons were surveyed for *Helicobacter* using fecal real‐time PCR. All baboons were positive for *Helicobacter* spp. with 78% positive for *H. macacae*, 19% positive for *H. suis* , 15% positive for *H. pylori* , and 0% positive for *H. cinaedi/canicola* (Table 1, Figure 2). Co‐infections were present in 30% of cases; all 4 animals positive for *H. pylori* were co‐infected with *H. macacae*, and 4 of the 5 animals positive for *H. suis* were co‐infected with *H. macacae*. The prevalence of *H. macacae* was similar between colony A (75%) and colony B (80%). There was no effect of colony or housing room on positivity with *H. macacae*, *H. suis* , or *H. pylori* (Table S4). There was an effect of captive‐born status on positivity with *H. macacae* (*Z* = −2.29, SE = 1.31, *p* = 0.022) with 87% (23/27) of captive‐born baboons positive for *H. macacae* compared to only 25% (1/4) of wild‐born baboons. Animals born at Facility 1 (*Z* = 2.46, SE = 1.55, *p* = 0.014) also had a significantly higher likelihood of positivity for *H. macacae* compared to wild‐born animals. The only hamadryas baboon in the study, baboon #20, tested positive for *H. macacae*; to our knowledge, this is the first report of *H. macacae* in *P. hamadryas* . There were no statistical effects of captive‐born status or birth facility on *H. suis* or *H. pylori* prevalence, nor of age or time at the facility on *H. macacae*, *H. suis* , or *H. pylori* prevalence.

**TABLE 1:** Initial fecal PCR results.

	ID	*Helicobacter* spp.	*H. macacae*	*H. suis*	*H. pylori*	*H. cinaedi*	Sequencing
Colony A	1	+	−	−	−	−	Atypical sequence
	2	+	−	−	−	−	Atypical sequence
	3	+	+	−	−	−	
	4	+	+	−	−	−	
	5	+	+	−	−	−	
	6	+	+	−	−	−	
	7	+	+	−	−	−	
	8	+	+	−	+	−	
	9	+	−	+	−	−	
	10	+	+	−	+	−	
	11	+	+	−	+	−	
	12	+	+	−	−	−	
Colony A prevalence		100%	75%	8%	25%	0%	
Colony B	13	+	−	−	−	−	Atypical sequence
	14	+	−	−	−	−	Atypical sequence
	15	+	+	+	−	−	
	16	+	+	+	−	−	
	17	+	+	−	−	−	
	18	+	+	−	+	−	
	19	+	−	−	−	−	Atypical sequence
	20	+	+	−	−	−	
	21	+	+	+	−	−	
	22	+	+	−	−	−	
	23	+	+	−	−	−	
	24	+	+	+	−	−	
	25	+	+	−	−	−	
	26	+	+	−	−	−	
	27	+	+	−	−	−	
Colony B prevalence		100%	80%	27%	7%	0%	
Overall prevalence		100%	78%	19%	11%	0%	

**FIGURE 2:**
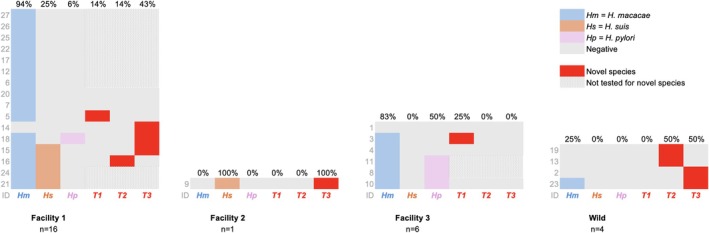
Prevalence of *Helicobacter* species by site of birth. A total of 27 baboons were screened for *Helicobacter macacae*, *H. suis* , *H. pylori* , and *H. cinaedi* by fecal PCR. Here, animals are categorized based on site of birth, with four animals wild‐born and the rest captive‐born. The *Helicobacter* PCR status of each animal, with identification (ID) numbers in gray, is indicated by a colored (positive) or gray (negative) box for *H. macacae* (*Hm*), *H. suis* (*Hs*), and *H. pylori* (*Hp*). No animals were positive for *H. cinaedi* . The prevalence of *H. macacae* was 94% (15/16) for baboons born at Facility 1, 0% (0/1) for Facility 2, 83% (5/6) for Facility 3, and 25% (1/4) for wild‐born baboons. Three of the four wild‐born baboons were negative for *H. macacae*, *H. suis* , and *H. pylori* . There was a significant effect of captive‐born status on *H. macacae* positivity (*Z* = −2.29, SE = 1.31, *p* = 0.022). Animals born at Facility 1 (*Z* = 2.46, SE = 1.55, *p* = 0.014) also had a significantly higher likelihood of *H. macacae* positivity than wild‐born animals. Coinfections were present in 30% (8/27) of cases. Sixteen baboons were screened for *Helicobacter* species by culture, preferentially selecting animals that were PCR‐negative for other *Helicobacter* spp. A total of 11/16 (69%) of animals cultured positive for a novel species (T1—*H. papionis*, T2—*H. simiae*, and T3—*H. papionifaecis*); none were positive for more than one novel species. Culture results are in red (positive), gray (negative) or dotted (no cultured sample).

### 3.3 Initial Identification of Novel *Helicobacter* Sequences

On initial colony survey, five animals (two from colony A, three from colony B) were positive for *Helicobacter* at the genus‐level but negative on species‐specific assays. Eight additional baboons in colony A met criteria after antibiotic treatment. Feces from these 13 animals were subjected to *16SrRNA ± 23SrRNA* and *gyrB* gene sequencing. Sequences from baboons #2 and #14 had 100% *16SrRNA* homology to *H. macacae* strains (MIT 99‐5501 and MIT 99‐5504) but only 93.3%–97.7% homology with *23SrRNA* sequences and 85.5%–86.7% homology with *gyrB* sequences from *H. macacae*. Feces from the other 11 baboons (#1, 3–10, 13, and 19) yielded *16SrRNA* sequences with > 99% identity with *H. fennelliae* (NCTC 11613), *H. cinaedi* Mainz strain (CCUG 33804/R‐927), baboon isolate MIT 03‐7674 colon, and macaque isolate MIT 99‐10781, but lower homology for *23SrRNA* and *gyrB* sequences. These results suggested the presence of undescribed species.

### 3.4 Treatment and Fecal PCR Follow‐Up

Due to potential adverse impacts of *Helicobacter* infection on experimental outcomes, one investigator elected to pursue treatment. Nine baboons from colony A were treated with oral triple therapy (amoxicillin (10 mg/kg), clarithromycin (10 mg/kg), and omeprazole (20 mg)) once daily for 10 days. Treatment was well tolerated. Fecal real‐time PCR performed 2 weeks post‐treatment demonstrated 100% clearance of *H. macacae*, *H. suis* , and *H. pylori* , yet eight of nine animals remained positive for *Helicobacter* at the genus level (Table 2). At 14–16 months post‐treatment, all baboons were positive for *Helicobacter* spp., but only two were identifiable at the species level (Table 2).

**TABLE 2:** Colony A, 2‐weeks post‐treatment fecal PCR results.

ID	Treatment	2‐weeks post‐treatment fecal PCR			14–16 month post‐treatment fecal PCR		
		*Helicobacter* spp.	*H. macacae*, *H. suis* , *H. pylori* , *H. cinaedi*	Sequencing	*Helicobacter* spp.	*H. suis*	*H. macacae*, *H. pylori* , *H. cinaedi*
1	X	−	−	*−*	(+)^a^	−	−
2	Not treated						
3	X	+	−	Atypical sequence	+	−	−
4	X	+	−	Atypical sequence	+	−	−
5	X	+	−	Atypical sequence	+	−	−
6	X	+	−	Atypical sequence	+	+	−
7	X	+	−	Atypical sequence	+	−	−
8	X	+	−	Atypical sequence	+	−	−
9	X	+	−	Atypical sequence	+	+	−
10	X	+	−	Atypical sequence	+	−	−
11	Not treated						
12	Not treated						
	Treatment efficacy	11%	100%		Reinfection rate in treated baboons	22%	0%

^a^ Positive signal was consistent with weak amplification near the limit of detection.

### 3.5 Case Study 2

Eight months following the initial *Helicobacter* diagnosis, baboon #14 from colony B presented for acute obtundation with marked gastric dilatation and was euthanized due to poor prognosis. Necropsy revealed chronic‐active gastritis and a tumor expanding and compressing the cystic bile duct and neck of the gall bladder, histologically consistent with a cholangiocarcinoma (Figure 3). Moderate periportal to bridging chronic hepatitis was also present, as well as mild to moderate chronic‐active enterocolitis. No organisms consistent with *Helicobacter* were appreciated on Warthin–Starry stained sections from the tumor, liver, gall bladder, stomach, small intestine, or colon. Additionally, scrolls of paraffin‐embedded tumor were negative for *Helicobacter* by *16S rRNA* PCR. However, *H. suis* was identified by PCR in feces and fresh tissue from necropsy (stomach, liver, gall bladder, small intestine and colon). The colon was also positive for *H. macacae*. Fecal PCR also yielded undescribed *Helicobacter* sequences identical to this animal's original sample.

**FIGURE 3:**
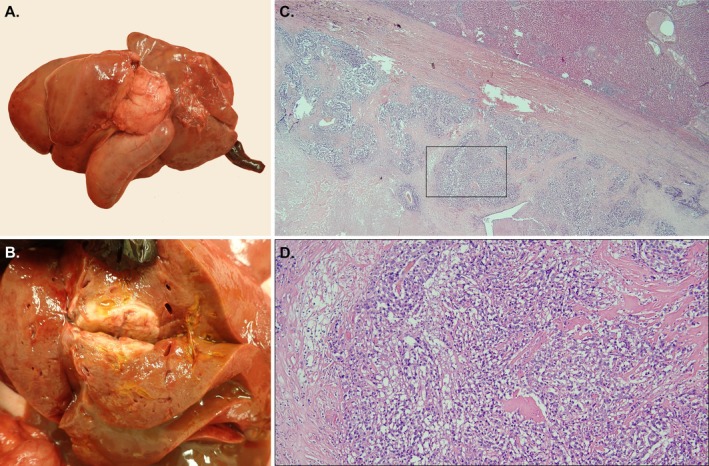
25‐year‐old male baboon ( *Papio anubis* ); extrahepatic cholangiocarcinoma. (A) Gross: Gall bladder distended approximately twofold with biliary sludge; approximately 4 cm diameter, pale, firm mass attached to and partially occluding the cystic bile duct and dorsally compressing the liver parenchyma. (B) On cut section, the mass consists of firm, tan to pink, heterogeneous tissue with central softening (necrosis) and yellow mucoid material. (C) Histology: Expanding the gall bladder wall and separated from the hepatic parenchyma by prominent fibrinocollagen, neoplastic polygonal cells are loosely arranged in nests and packets, with prominent fibrous stroma (desmoplasia) and areas of necrosis. In adjacent liver, there is portal‐periportal inflammation and fibrosis and central vein fibrosis. H&E. Magnification: 20×. (D) Neoplastic cell population, H&E. Magnification: 100×.

### 3.6 Isolation and Characterization of Three Novel *Helicobacter* Species

Approximately 22 months (Colony A) and 8 months (Colony B) after the initial survey, fecal samples were cultured from 16 baboons (seven in Colony A and nine in Colony B). Samples that tested positive for *Helicobacter* spp. but negative for *H. macacae* were preferentially chosen for culture. A total of 11 *Helicobacter* isolates were obtained (two in Colony A and nine in Colony B). Sequencing of the *16S rRNA*, *hsp60* and *rpoB* genes and phylogenetic analysis revealed three novel taxa that clustered separately from other *Helicobacter* species (Figure S1). The presence of three novel taxa was subsequently confirmed by whole genome sequencing of two isolates per novel taxon, for which we propose the names *H. papionis*, *H. simiae*, and *H. papionifaecis* (Figure 4). *H. simiae* and *H. papionifaecis* were phylogenetically closer to each other than to *H. papionis. H. papionifaecis* was closely related to *H. macacae*. Average nucleotide identity (ANI) calculated between novel and existing *Helicobacter* genomes revealed that the novel species were below the 95% similarity threshold to differentiate species, and digital DNA–DNA hybridization (DDH) showed that the novel species were below the 70% similarity threshold to differentiate species (Table S5). Thus, we confirmed that the three novel species are distinct from each other and from other known *Helicobacter* species. Genome summary statistics are presented in Table S6. *H. papionifaecis* had a substantially larger genome than the other two novel species, at 2.78 and 2.89 Mb for isolates 23‐1056 and 23‐1059, respectively. All sequenced isolates contained the gene encoding the virulence factor flagellin, and all but 23‐1046 T2 (*H. simiae*) contained the gene encoding catalase. The novel species were negative for other major virulence factors, including CagA. The *16S rRNA*, *23S rRNA*, and *gyrB* sequences from initial characterization were also evaluated using BLAST against whole genome sequences from pure isolates to confirm identity.

**FIGURE 4:**
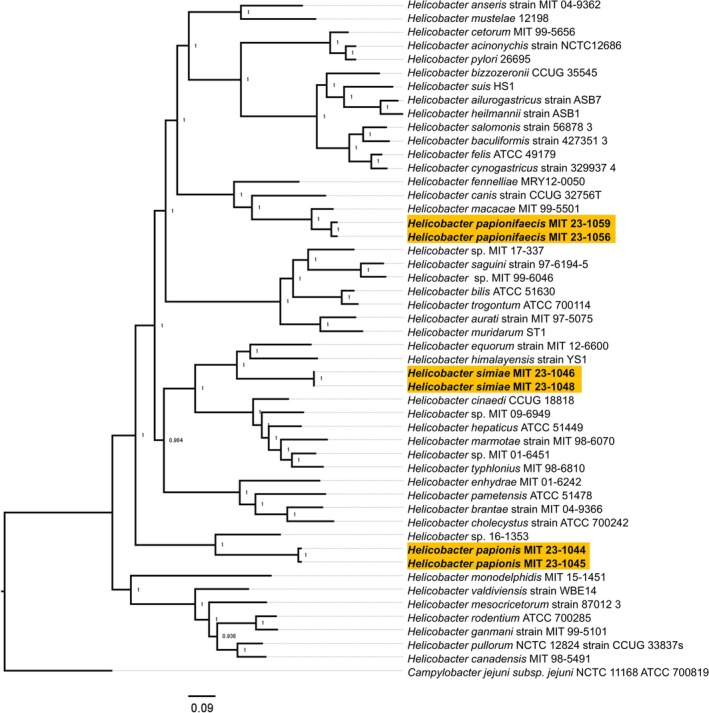
Whole genome phylogeny. Whole‐genome phylogenetic tree constructed from multi‐sequence alignment of concatenated core protein sequences. Local support values are indicated on the branches. Genomes from type strains were used when available.

A comparison of the biochemical and phenotypic characterizations of the three novel species and other *Helicobacter* species is provided in Table S7. All isolates were oxidase‐positive, urease‐negative, negative for γ‐glutamyl transferase activity, positive for alkaline phosphatase hydrolysis, negative for nitrate reduction to nitrite, and unable to grow in 1% glycine but able to grow at 42°C. *H. papionis* and *H. papionifaecis* were catalase‐positive, while *H. simiae* was catalase‐negative. Indoxyl acetate hydrolysis was present in all isolates of *H. papionis*, one of five isolates of *H. simiae*, and two of four isolates of *H. papionifaecis*. All three novel species were resistant to the antibiotic nalidixic acid, while *H. simiae* and *H. papionifaecis* were also resistant to cephalothin. Transmission electron microscopy was performed for two isolates from each novel species, with representative images from one isolate each shown in Figure 5. Cells from *H. papionis* were 1.2–1.5 μm long and 0.3–0.4 μm wide. The organism was comma‐shaped with a single, unipolar, sheathed flagellum. Cells from *H. simiae* were 2.6–4.3 μm long and 0.2–0.3 μm wide. The organism had curved to spiral shape with bipolar, sheathed flagella. Cells from *H. papionifaecis* were 3.1–4.4 μm long and 0.2–0.4 μm wide. The organism was curved with bipolar, sheathed flagella. No organisms had periplasmic fibers.

**FIGURE 5:**
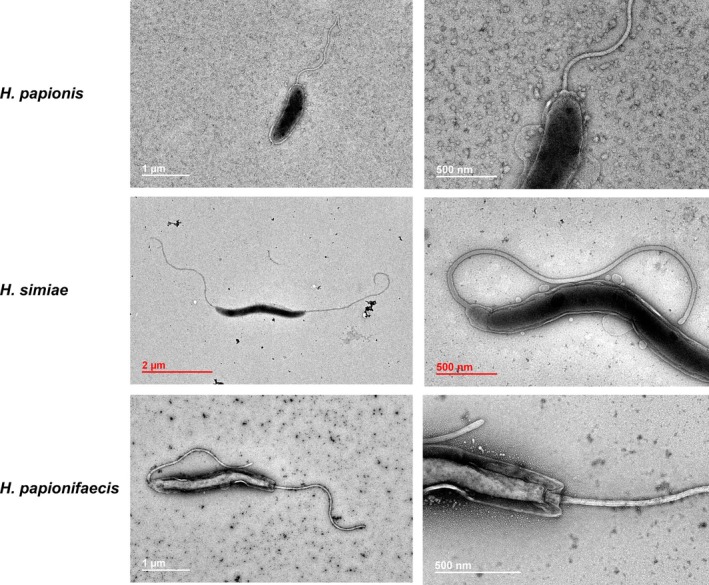
Transmission electron microscopy. Representative transmission electron microscopy images of the novel baboon *Helicobacter* isolates.

## 4 Discussion

This is the first colony‐wide survey of *Helicobacter* infection in baboons using molecular diagnostics and the first study to isolate novel *Helicobacter* spp. from baboons. Using a combination of genus‐level *Helicobacter* real‐time PCR and species‐specific real‐time PCR assays, we found that *H. macacae* is highly prevalent in our baboon colonies, with *H. suis* and *H. pylori* at lower prevalences. Thirteen baboons tested positive for *Helicobacter* spp. but negative on four species‐specific real‐time PCR assays, suggesting that they might be infected with species or strains of *Helicobacter* spp. that had not been previously identified in NHPs. Further investigation using culture and molecular diagnostics led to the isolation and characterization of three novel *Helicobacter* species: *H. papionis*, *H. simiae*, and *H. papionifaecis*. These species were genetically, morphologically, and biochemically distinct. All three species were resistant to the synthetic quinolone nalidixic acid, and *H. simiae* and *H. papionifaecis* were resistant to cephalothin, a first‐generation cephalosporin. The heterogeneity in antibiotic susceptibility observed among these novel *Helicobacter* species suggests that genus‐level identification of *Helicobacter* should be followed by species‐specific characterization to guide antibiotic therapy.

*H. macacae*, *H. suis* , and *H. pylori* were not cultured from any fecal samples, including baboons that were PCR‐positive for these species. This is in part due to intentional selection of samples for culture that were suspected to be mono‐infected by novel species. Additionally, because the samples for PCR and culture were collected at different times, intermittent shedding of the pathogen could impact test congruency. Furthermore, the ability to detect gastric species of *Helicobacter* by fecal PCR may be limited by their tissue niche deep in the gastric glands; thus, gastric biopsy with culture would be more sensitive; due to invasiveness, we did not employ this method here. The greater sensitivity of PCR compared to culture and the commercial availability of PCR assays led us to use PCR to assess treatment efficacy against *Helicobacter* in this study.

There are no studies on treatment for *Helicobacter* spp. in baboons, nor for EHS in any NHPs. Oral combinations of a proton‐pump inhibitor, amoxicillin, and clarithromycin, with or without bismuth subsalicylate, have been utilized successfully to treat gastric *Helicobacter* ( *H. pylori* in humans, rhesus macaques, and Japanese macaques, and *H. suis* in a rhesus macaque) [8, 43, 44, 45, 46]. In rhesus macaques, *H. pylori* has also been managed with metronidazole, amoxicillin, and bismuth subsalicylate, but with only partial success due to metronidazole resistance [43]. There is sparse guidance on the treatment of EHS in human patients. Intravenous antibiotic therapy for several weeks appears to be effective in human clinical reports, but such regimens would often be impractical to implement for NHPs, and difficult to justify in animals without clinical signs [6]. In our study, oral triple therapy with once daily amoxicillin, clarithromycin, and omeprazole was safe and effective to treat *H. macacae*, *H. pylori* , and *H. suis* in baboons. Animals remained negative for *H. macacae* and *H. pylori* by fecal PCR for over 1 year, suggestive of clearance or long‐term suppression. Despite initial 2‐week post‐treatment negative results, two baboons later became positive for *H. suis* , which may be new infections or recrudescence. The successful treatment of *H. macacae* is particularly informative for primate veterinarians.

Further research is indicated to investigate how baboons acquire these apparently prevalent *Helicobacter* spp. infections. *H. papionis* was only found in Colony A, while *H. simiae* was restricted to Colony B and *H. papionifaecis* was predominantly found in Colony B, with one individual positive in Colony A. We found no correlation between species‐specific *Helicobacter* positivity and colony or housing room, suggesting that transmission among adult baboons may not be common. Despite a small sample size, we also found that three of the four wild‐born animals were negative for *H. macacae*, *H. suis* , and *H. pylori* , despite having been at our institution for over 14 years and with other baboons housed in proximity. In captive rhesus macaques, newborns are infected with *H. pylori* via oral‐oral exposure within 24 h of life [47]. Host adaptation to *H. pylori* also appears to play a role in macaques [48]. While EHS have been less studied in NHPs, chronic subclinical infections with *H. macacae* and *H. cinaedi* may be common in macaques [13, 49].

Zoonotic or zooanthroponotic transmission of *Helicobacter* between humans and NHPs may also occur [50, 51, 52]. Further characterization of natural *Helicobacter* infections in baboons is thus also essential for biosafety and risk assessment with regard to personnel exposure.

On necropsy, we identified subclinical pathology of the hepatobiliary and/or gastrointestinal systems in 4/6 animals, including a cholangiocarcinoma in baboon #14, representing the first such case documented in an olive baboon. Gall bladder adenocarcinomas have been rarely reported in baboons, with three cases in hamadryas baboons, three in Guinea baboons ( *Papio papio* ), and no reports in several large retrospective studies [53, 54, 55, 56, 57, 58, 59]. Although *Helicobacter* was not identified within the tumor microscopically or by PCR, this baboon was PCR‐positive for *H. suis* and *H. macacae* in feces and tissues, and positive for *H. simiae* by fecal culture. Since EHS are associated with hepatobiliary tumors in humans, chronic *Helicobacter* spp. infection in this baboon could have participated in tumorigenesis [60, 61, 62, 63, 64, 65]. Further investigation of the potential sequelae of chronic *Helicobacter* infection in baboons is therefore needed to inform clinical management.

Given the apparently high prevalence and chronicity of *Helicobacter* infection in predominantly healthy baboons, we hypothesize that these organisms may be commensals or pathobionts. We also suspect that conspecific transmission may occur in baboons with isolated colonies having distinct *Helicobacter* species or strains, but that transmission among adult baboons may not be very common and may be influenced by other factors which merit further study—for example, age of first exposure, coinfection with other *Helicobacter* strains, or health status. Triple therapy treatment achieved prolonged clearance of *H. macacae* and *H. pylori* . This is the first published treatment protocol for *Helicobacter* in baboons and represents an evidence‐based refinement to the management of this species. However, triple therapy treatment was ineffective at clearing the novel *Helicobacter* species, and isolates showed antibiotic resistance in vitro, suggesting the response to treatment is likely bacterial species‐specific and highlighting the value of species‐specific assays to guide management and therapy.

## Funding

This work was supported by Johns Hopkins University Research Animal Resources, IDEXX BioAnalytics, and NIH R35 CA210088 and 1R01 CA281732‐01 (to J.G.F.). Training support for I.A.J. was provided by NIH T32 OD011089.

## Conflicts of Interest

M.J.C. and S.L.P. are employees of IDEXX BioAnalytics, a division of IDEXX Laboratories Inc., a company that provides veterinary diagnostics. The other authors declare no conflicts of interest.

## Supporting information


**Figure S1:** Phylogenetic analysis based on Sanger Sequencing of *16S rRNA*, *hsp60*, and *rpoB* gene sequences.
**Table S1:** Infections with *Helicobacter* species documented in olive baboons ( *Papio anubis* ) and hamadryas baboons ( *P. hamadryas* ).
**Table S2:** Demographic data and medical history of baboons.
**Table S3:** Forward and reverse primer sequences for PCR amplification of *16SrRNA*, *23SrRNA*, *gyrB*, *hsp60*, and *rpoB* target genes.
**Table S4:** Statistical analysis of potential risk factors associated with *Helicobacter* spp. infection in baboons.
**Table S5:** Average nucleotide identity (ANI) and digital DNA–DNA hybridization (dDDH) comparisons of novel *Helicobacter* isolates.
**Table S6:** Genome summary statistics for novel *Helicobacter* strains isolated from baboons.
**Table S7:** Phenotypic characteristics that differentiate these three novel taxa from other *Helicobacter* species.

## Data Availability

The data that support the findings of this study are available from the corresponding authors upon reasonable request.
