# Supplementary material for: Clinical Management of Helicobacter Infection in Captive Baboons ( Papio anubis and Papio hamadryas ), and Isolation and Molecular Characterization of Three Novel Helicobacter Species
Source: J Med Primatol. 2026 Jun 19;55(4):e70095. doi: 10.1111/jmp.70095 (PMC13280779; doi:10.1111/jmp.70095)
Supplement: Supplementary file 1 — Figure S1: Phylogenetic analysis based on Sanger Sequencing of 16S rRNA, hsp60, and rpoB gene sequences. Table S1: Infections with Helicobacter species documented in olive baboons ( Papio anubis ) and hamadryas baboons ( P. hamadryas ). Table S2: Demographic data and medical history of baboons. Table S3: Forward and reverse primer sequences for PCR amplification of 16SrRNA, 23SrRNA, gyrB, hsp60, and rpoB target genes. Table S4: Statistical analysis of potential risk factors associated with Helicobacter spp. infection in baboons. Table S5: Average nucleotide identity (ANI) and digital DNA–DNA hybridization (dDDH) comparisons of novel Helicobacter isolates. Table S6: Genome summary statistics for novel Helicobacter strains isolated from baboons. Table S7: Phenotypic characteristics that differentiate these three novel taxa from other Helicobacter species. [file JMP-55-e70095-s001.docx]

**Clinical management of *Helicobacter* in captive baboons (*Papio* *anubis* and *Papio hamadryas*), and isolation and molecular characterization of three novel *Helicobacter* species**

**
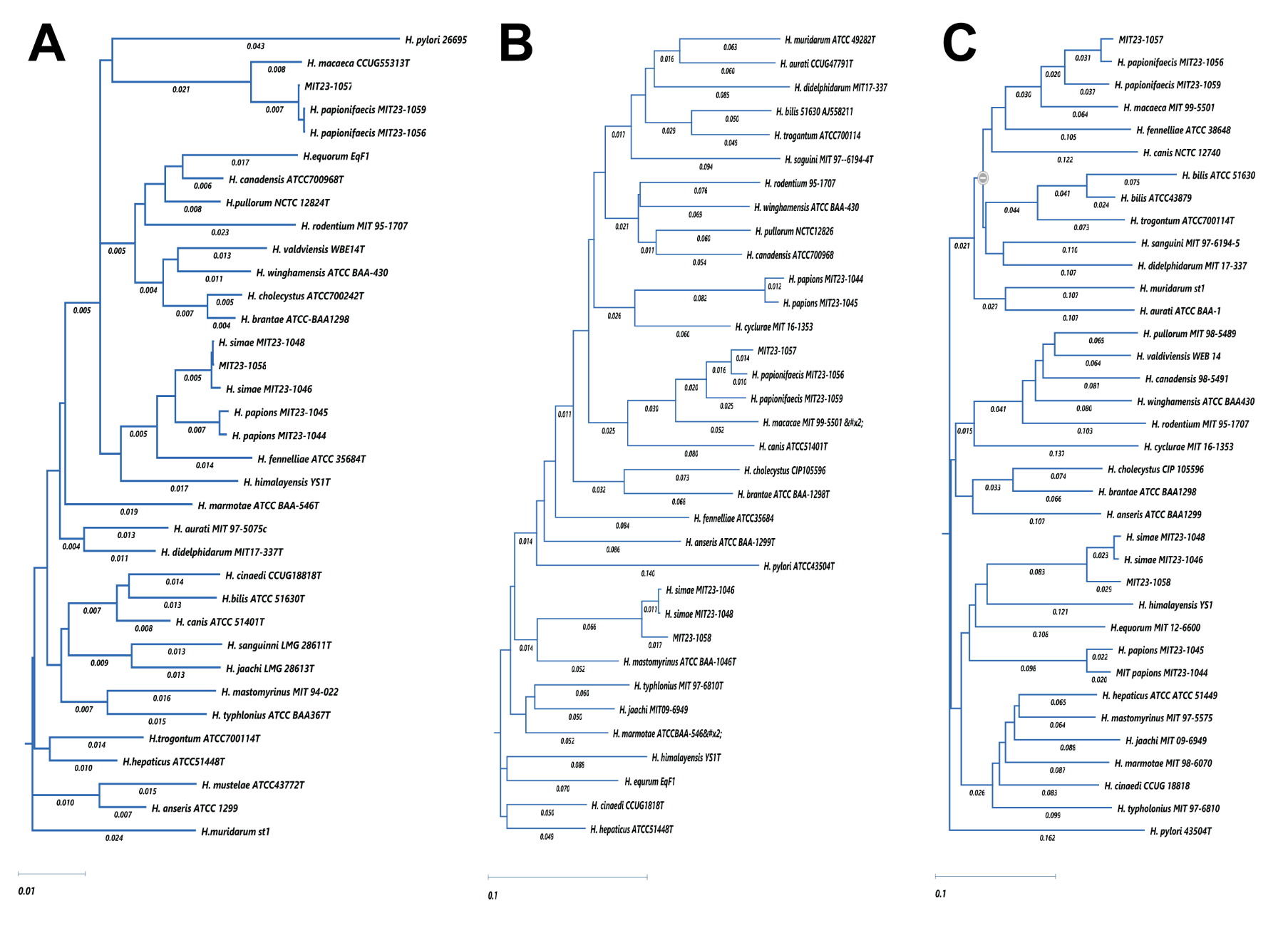
**

**Supplemental Figure 1.** Phylogenetic analysis based on Sanger Sequencing of *16S rRNA* **(A)**, *hsp60* **(B)**, and *rpoB* **(C)** gene sequences. Genes from different *Helicobacter* species were used to form neighbor-joining trees. Bars indicate the number of nucleotide substitutions.

**Supplemental Table 1:** Infections with *Helicobacter* species documented in olive baboons (*Papio anubis*) and hamadryas baboons (*P. hamadryas*).

| ***Helicobacter* species** | **Isolate name** | **Host species** | **Body site** | **Method of identification and reference** |
| --- | --- | --- | --- | --- |
| *H. pylori* | *-* | *Papio* spp. | Stomach | 16SrRNA sequencing^1^  Antibody detection, morphology, biochemical testing^2^  Method not specified^3^ |
| *H. pylori* | *-* | *Papio hamadryas* | Stomach | Morphology, 16SrRNA and urease gene sequencing^4^ |
| HHLOs | *-* | *Papio* spp. | Stomach | Morphology, 16SrRNA and urease gene sequencing^4^  Antibody detection, morphology, biochemical testing^2^  Morphology, biochemical testing^5^ |
| HHLOs | *-* | *Papio* *anubis* | Stomach | Morphology^6,7^ |
| *H. heilmannii* | *-* | *Papio hamadryas* | Stomach | 16SrRNA sequencing, morphology^8^ |
| *H. macacae* (MIT 99-5501, MIT 99-5504) | MIT 03-7674-liver | *Papio anubis* | Liver | *16SrRNA* sequencing^9^ |
| *H. macacae* (MIT 99-5501, MIT 99-5504) | MIT 03-7674-ileum | *Papio anubis* | Ileum | *16SrRNA* sequencing^9^ |
| *H. cinaedi,* Mainz strain (CCUG 33804, R-927) | MIT 03-7674-colon | *Papio anubis* | Colon | *16SrRNA* sequencing^9^ |
| *H. cinaedi*, Mainz strain (CCUG 33804, R-927) | - | *Papio hamadryas* | Feces | *16SrRNA* sequencing^10^ |

**Supplemental Table 2:** Demographic data and medical history of baboons.

| **ID** | **Colony** | **Room** | **Sex** | **Species** | **Age (years)** | **Tenure (years)** | **Prior facility** | **Inter-mediate facility** | **Site of birth** | **Treatment** | **Reaction to treatment** | **Acute GI Hx** | **Chronic GI Hx** | **Major Medical Hx** | **Euthanasia** | **Necropsy** |
| --- | --- | --- | --- | --- | --- | --- | --- | --- | --- | --- | --- | --- | --- | --- | --- | --- |
| 1 | A | A1 | M | *P. anubis* | 16.7 | 13.4 | Facility 3 | None | Facility 3 | 1 | 0 | 1 | 0 | 0 | 1 | 1 |
| 2 | A | A1 | M | *P. anubis* | 19.5 | 17.0 | Facility 6 | None | Wild | 0 |  | 1 | 0 | 0 | 1 | 1 |
| 3 | A | A1 | M | *P. anubis* | 15.4 | 11.9 | Facility 3 | None | Facility 3 | 1 | 0 | 1 | 0 | 0 | 1 | 1 |
| 4 | A | A1 | M | *P. anubis* | 15.4 | 11.9 | Facility 3 | None | Facility 3 | 1 | 1 | 0 | 0 | 0 | 0 |  |
| 5 | A | A1 | M | *P. anubis* | 19.7 | 14.3 | Facility 1 | None | Facility 1 | 1 | 0 | 0 | 0 | 0 | 0 |  |
| 6 | A | A1 | M | *P. anubis* | 20.4 | 14.3 | Facility 1 | None | Facility 1 | 1 | 0 | 0 | 0 | 0 | 1 | 1 |
| 7 | A | A1 | M | *P. anubis* | 23.4 | 17.9 | Facility 1 | None | Facility 1 | 1 | 0 | 0 | 0 | 0 | 1 | 1 |
| 8 | A | A1 | M | *P. anubis* | 7.2 | 3.3 | Facility 3 | None | Facility 3 | 1 | 0 | 0 | 0 | 0 | 0 |  |
| 9 | A | A1 | M | *P. anubis* | 17.7 | 13.4 | Facility 3 | None | Facility 2 | 1 | 0 | 1 | 0 | 0 | 0 |  |
| 10 | A | A2 | M | *P. anubis* | 7.1 | 3.1 | Facility 3 | None | Facility 3 | 1 | 0 | 0 | 0 | 0 | 1 | 1 |
| 11 | A | A2 | M | *P. anubis* | 17.3 | 12.0 | Facility 3 | None | Facility 3 | 0 |  | 1 | 0 | 0 | 1 | 0 |
| 12 | A | A2 | F | *P. anubis* | 3.6 | 1.9 | Facility 4 | None | Facility 1 | 0 |  | 0 | 0 | 0 | 0 |  |
| 13 | B | B2 | M | *P. anubis* | 19.4 | 18.4 | Facility 6 | None | Wild | 0 |  | 0 | 0 | 0 | 0 |  |
| 14 | B | B2 | M | *P. anubis* | 24.3 | 14.0 | Facility 7 | None | Facility 1 | 0 |  | 1 | 0 | 0 | 1 | 1 |
| 15 | B | B2 | M | *P. anubis* | 19.3 | 14.4 | Facility 1 | None | Facility 1 | 0 |  | 0 | 0 | 0 | 0 |  |
| 16 | B | B2 | M | *P. anubis* | 21.4 | 14.2 | Facility 7 | None | Facility 1 | 0 |  | 0 | 0 | 0 | 0 |  |
| 17 | B | B2 | M | *P. anubis* | 19.8 | 14.4 | Facility 1 | None | Facility 1 | 0 |  | 0 | 0 | 0 | 0 |  |
| 18 | B | B2 | M | *P. anubis* | 26.3 | 20.6 | Facility 1 | None | Facility 1 | 0 |  | 1 | 0 | 0 | 0 |  |
| 19 | B | B2 | M | *P. anubis* | 23.1 | 21.9 | Facility 8 | None | Wild | 0 |  | 1 | 0 | 0 | 0 |  |
| 20 | B | B1 | M | *P. hamadryas* | 24.8 | 19.5 | Facility 1 | None | Facility 1 | 0 |  | 0 | 0 | 0 | 0 |  |
| 21 | B | B1 | M | *P. anubis* | 3.9 | 2.0 | Facility 4 | Facility 1 | Facility 1 | 0 |  | 0 | 0 | 0 | 0 |  |
| 22 | B | B1 | M | *P. anubis* | 3.9 | 2.0 | Facility 4 | Facility 1 | Facility 1 | 0 |  | 0 | 0 | 0 | 0 |  |
| 23 | B | B1 | M | *P. anubis* | 20.5 | 14.2 | Facility 5 | Facility 6 | Wild | 0 |  | 0 | 0 | 0 | 0 |  |
| 24 | B | B1 | M | *P. anubis* | 26.7 | 21.4 | Facility 1 | None | Facility 1 | 0 |  | 0 | 0 | 0 | 0 |  |
| 25 | B | B1 | M | *P. anubis* | 24.2 | 16.2 | Facility 1 | None | Facility 1 | 0 |  | 0 | 0 | 0 | 0 |  |
| 26 | B | B1 | M | *P. anubis* | 25.1 | 21.1 | Facility 1 | None | Facility 1 | 0 |  | 0 | 0 | 0 | 0 |  |
| 27 | B | B1 | M | *P. anubis* | 27.3 | 21.6 | Facility 1 | None | Facility 1 | 0 |  | 0 | 0 | 0 | 0 |  |

**Supplemental Table 3:** Forward and reverse primer sequences for PCR amplification of *16SrRNA*, *23SrRNA*, *gyrB*, *hsp60*, and *rpoB* target genes.

| Gene and sample | Primer sequences |
| --- | --- |
| *16SrRNA*; fecal samples | Forward 5’-CTATGACGGGTATCCGGC -3’  Reverse 5’- ATTCCACCTACCTCTCCCA-3’ |
| *23SrRNA*; fecal samples | Forward 5’-AGGACGTACTAGACTGCGATAAGCTAT-3’  Reverse 5’- GTACTTGTTCGCTATCGGTGTGA-3’ |
| *gyrB*; fecal samples | Forward 5’-AGAGATGGCACGGAGGTAAGC-3’  Reverse 5’-GCAGAGGCAAAATCGCTTG-3’ |
| *16S rRNA*; paraffin-embedded tumor | C97 Forward 5'-GCTATGACGGGTATCC-3'  H676 Reverse 5`-ATTCCACCTACCTCTCCCA-3` and C98N Reverse 5'-GATTTTACCCCTACACCA-3' |
| *16SrRNA* (pure isolates) | 9F 5′-GAGTTTGATYCTGGCTCAG-3′  1541R 5′-AAGGAGGTGWTCCARCC-3′ |
| *hsp60* (pure isolates) | HSP60AF 5′-GCTAATCCTATTGAAGTGAAAAGAGGNATGGAYAA-3′  HSP60DR 5′-CACTAAGGTAGTTAAAGCTTCCCCTTCDATRTCYT-3′ |
| *rpoB* (pure isolates) | rpoB-F 5’-GGDCARCTYTCNCARTTYATGG-3’  He-rpoB-R 5’-GYTGCATRTTWGWHCCCAT-3’ |

**Supplemental Table 4:** Statistical analysis of potential risk factors associated with *Helicobacter* spp. infection in baboons.

|  | Variable | *H. macacae* | *H. suis* | *H. pylori* |
| --- | --- | --- | --- | --- |
| Age | Years | Z = 1.32, SE = 0.28, P = 0.188 | Z = 0.30 SE = 0.24, P = 0.762 | Z = 0.86 SE = 0.30, P = 0.396 |
| Tenure | Years | Z = -1.59, SE = 0.31, P = 0.112 | Z = -0.36, SE = 0.29, P = 0.719 | Z = -1.10, SE = 0.38, P = 0.273 |
| GI signs | Presence vs Absence | Z = 0.94, SE = 1.11, P = 0.347 | Z = -0.52, SE = 1.21, P = 0.605 | Z = -0.94, SE = 1.11, P = 0.347 |
| Colony | Colony A vs Colony B | Z = -0.31, SE = 0.93, P = 0.757 | Z = -1.60, SE = 1.20.85, P = 0.247 | Z = 1.25, SE = 1.23, P = 0.211 |
| Room | Room A1 vs Room A2 | Z = 0.00, SE = 6208.83, P = 0.998 | Z = -0.00, SE = 3765.84, P = 0.997 | Z = 1.71, SE = 1.62, P = 0.087 |
|  | Room A1 vs Room B1 | Z = 0.00, SE = 3802.12, P = 0.996 | Z = 0.73, SE = 1.34, P = 0.464 | Z = -0.01, SE = 3802.12, P = 0.996 |
|  | Room A1 vs Room B2 | Z = -0.39, SE = 1.04, P = 0.697 | Z = 0.86, SE = 1.35, P = 0.389 | Z = 0.19, SE = 1.51, P = 0.849 |
|  | Room B1 vs Room B2 | Z = -0.00, SE < 0.001, P = 0.996 | Z = 0.16, SE = 1.17, P = 0.876 | Z = 0.01, SE = 3802.12, P = 0.996 |
| Wild born | Captive born vs Wild | **Z = -2.29, SE = 1.31, P = 0.022** | Z = -0.01, SE = 3261.32, P = 0.996 | Z = -2.83, SE = 3261.32, P = 0.996 |
| Birth Site | Facility 1 vs Wild | **Z = 2.46, SE = 1.55, P = 0.014** | Z = 0.00, SE < 0.001, P = 0.997 | Z = 0.01, SE < 0.001, P = 0.997 |
|  | Facility 2 vs Wild | Z = -0.00, SE = 3956.18, P = 0.997 | Z = 0.00, SE < 0.001, P = 0.997 | Z = 0.00, SE < 0.001, P = 1.00 |
|  | Facility 3 vs Wild | Z = 1.70, SE = 1.59, P = 0.089 | Z = 0.00, SE < 0.001, P = 1.00 | Z = 0.01, SE < 0.001, P = 0.995 |
| Prior Facility | Facility 3 vs Facility 1 | Z = -0.00, SE < 0.001, P = 0.997 | Z = -0.22, SE = 1.33, P = 0.829 | Z = 1.55, SE = 1.30, P = 0.120 |
|  | Facility 4 vs Facility 1 | Z = -0.00, SE < 0.001, P = 1.000 | Z = 0.558, SE = 1.45, P = 0.577 | Z = -0.00, SE = 6208.83, P = 0.998 |
|  | Facility 5 vs Facility 1 | Z = 0.00, SE < 0.001, P = 1.000 | Z = -0.00, SE = 6522.64, P = 0.998 | Z = -0.00, SE = 10754.01, P = 0.999 |
|  | Facility 6 vs Facility 1 | Z = -0.00, SE < 0.001, P = 0.998 | Z = -0.00, SE = 4612.20, P = 0.997 | Z = -0.00, SE = 7604.24, P = 0.998 |
|  | Facility 7 vs Facility 1 | Z = -0.00, SE < 0.001, P = 0.997 | Z = 0.00, SE < 0.001, P = 0.352 | Z = -0.00, SE = 7604.24, P = 0.998 |
|  | Facility 8 vs Facility 1 | Z = -0.00, SE < 0.001, P = 0.999 | Z = -0.00, SE = 6522.64, P = 0.998 | Z = -0.00, SE = 10754.01, P = 0.999 |

**Supplemental Table 5.** Average nucleotide identity (ANI) (Upper) and digital DNA-DNA hybridization (dDDH) (Lower) comparisons of novel *Helicobacter* isolates. All ANI and DDH comparisons were below the 95 and 70% thresholds, respectively, indicating *H. papionis*, *H. simiae*, and *H. papionifaecis* are each novel species.

| **ANI** | **23-1044**  ***H. papionis*** | | **23-1045**  ***H. papionis*** | | | **23-1046**  ***H. simiae*** | | **23-1048**  ***H. simiae*** | **23-1056**  ***H. papionifaecis*** | | | **23-1059**  ***H. papionifaecis*** |  |
| --- | --- | --- | --- | --- | --- | --- | --- | --- | --- | --- | --- | --- | --- |
| **23-1044**  ***H. papionis*** | **100.0** | | **94.7** | | | **75.4** | | **76.2** | **76.5** | | | **75.9** |  |
| **23-1045**  ***H. papionis*** | **94.7** | | **100.0** | | | **76.1** | | **76.6** | **76.5** | | | **75.5** |  |
| **23-1046**  ***H. simiae*** | **75.3** | | **76.2** | | | **100.0** | | **98.4** | **81.0** | | | **86.1** |  |
| **23-1048**  ***H. simiae*** | **75.1** | | **75.9** | | | **98.3** | | **100.0** | **80.8** | | | **85.1** |  |
| **23-1056**  ***H. papionifaecis*** | **75.8** | | **76.1** | | | **80.4** | | **80.9** | **100.0** | | | **88.3** |  |
| **23-1059**  ***H. papionifaecis*** | **75.2** | | **75.1** | | | **85.5** | | **85.2** | **88.6** | | | **100.0** |  |
| **dDDH** | | **23-1044**  ***H. papionis*** | | **23-1045**  ***H. papionis*** | **23-1046**  ***H. simiae*** | | **23-1048**  ***H. simiae*** | | | **23-1056**  ***H. papionifaecis*** | **23-1059**  ***H. papionifaecis*** | | |
| **23-1044**  ***H. papionis*** | | **100.0** | | **58.4** | **31.1** | | **30.2** | | | **31.4** | **29.2** | | |
| **23-1045**  ***H. papionis*** | | **58.4** | | **100.0** | **29.3** | | **28.2** | | | **29.4** | **28.2** | | |
| **23-1046**  ***H. simiae*** | | **31.1** | | **29.3** | **100.0** | | **84.2** | | | **32** | **46.9** | | |
| **23-1048**  ***H. simiae*** | | **30.2** | | **28.2** | **84.2** | | **100.0** | | | **31.6** | **44.2** | | |
| **23-1056**  ***H. papionifaecis*** | | **31.4** | | **29.4** | **32** | | **31.6** | | | **100.0** | **35.2** | | |
| **23-1059**  ***H. papionifaecis*** | | **29.2** | | **28.2** | **46.9** | | **44.2** | | | **35.2** | **100.0** | | |

**Supplemental Table 6.** Genome summary statistics for novel *Helicobacter* strains isolated from baboons.

| **Species** | **MIT ID** | **Contigs** | **Size (bp)** | **GC Content** | **Contig L50** | **Contig N50** | **tRNA** | **tRNA** | **Protein Genes (CDS)** | **Virulence Factors** | **GenBank Accession** |
| --- | --- | --- | --- | --- | --- | --- | --- | --- | --- | --- | --- |
| *H. papionis* | 23-1044 | 43 | 2010198 | 41.05 | 5 | 131358 | 36 | 2 | 2373 | Flagellin, catalase | [JBSIUQ000000000](https://www.ncbi.nlm.nih.gov/nuccore/JBSIUQ000000000) |
| *H. papionis* | 23-1045 | 42 | 2005507 | 40.99 | 4 | 189078 | 36 | 2 | 2343 | Flagellin, catalase | [JBSIUR000000000](https://www.ncbi.nlm.nih.gov/nuccore/JBSIUR000000000) |
| *H. simiae* | 23-1046 | 10 | 1861768 | 40.23 | 1 | 1319148 | 37 | 2 | 1869 | Flagellin | [JBSIUS000000000](https://www.ncbi.nlm.nih.gov/nuccore/JBSIUS000000000) |
| *H. simiae* | 23-1048 | 3 | 1859658 | 40.24 | 1 | 1553674 | 36 | 2 | 1862 | Flagellin, catalase | [JBSIUT000000000](https://www.ncbi.nlm.nih.gov/nuccore/JBSIUT000000000) |
| *H. papionifaecis* | 23-1056 | 118 | 2781652 | 39.09 | 10 | 95611 | 38 | 2 | 3338 | Flagellin, catalase | [JBSIUU000000000](https://www.ncbi.nlm.nih.gov/nuccore/JBSIUU000000000) |
| *H. papionifaecis* | 23-1059 | 274 | 2893036 | 38.14 | 18 | 51408 | 37 | 2 | 3363 | Flagellin, catalase | [JBSIUV000000000](https://www.ncbi.nlm.nih.gov/nuccore/JBSIUV000000000) |

**Supplemental Table 7**: Phenotypic characteristics that differentiate these three novel taxa from other *Helicobacter* species.

Species: 1, *H. papionis*; 2, *H. simiae*; 3,  *H. papionifaecis*; 4, *H. monodelphidis*; 5, *H. didelphidarum*; 6, *H. acinonychis*; 7, *H. ailurogastricus*; 8, *H. anseris*; 9, *H. apri*; 10, *H. aurati*; 11, *H. baculiformis*; 12, *H. bilis*; 13, *H. bizzozeronii*; 14, *H. brantae*; 15, *H. canadensis*; 16, *H. canis*; 17, *H. cetorum*; 18, *H. cholecystus*; 19, *H. cinaedi*; 20, *H. cynogastricus*; 21, *H. equorum*; 22, *H. felis*; 23, *H. fennelliae*; 24, *H. ganmani*; 25, *H. heilmannii*; 26, *H. hepaticus*; 27, *H. himalayensis*; 28, *H. jaachi*; 29, *H. japonicus*; 30, *H. macacae*; 31, *H. marmotae*; 32, *H. mesocricetorum*; 33, *H. muridarum*; 34, *H. mustelae*; 35, *H. pametensis*; 36, *H. pullorum*; 37, *H. pylori*; 38, *H. rodentium*; 39, *H. saguini*; 40, *H. salomonis*; 41, *H. suis*; 42, *H. trogontum*; 43, *H. typhlonius*; 44, *H. valdiviensis*; 45, *H. cyclurae*; 46, *H. gastrocanis*; 47, *H. gastrofelis*; 48, *H. felistomachi*; 49, *H. anatolicus*; 50, *H. kayseriensis*; 51, *H. cappadocius*; 52, *H. colisuis*; 53, *H. ibis*; 54, *H. kumamotonensis*; 55, *H. vulpis*; 56, *H. mehlei*; 57, *H. labacensis*; 58, *H. turcicus*; 59, *H. zhangjianzhongii*; 60, *H. delphincola*; +, All strains examined give a positive result; −, all strains examined give a negative result; (+), 80–94 % strains positive; ±, 33–66 % strains positive; (−), 7–33 % strains positive; NA, nalidixic acid; CE, Cephalothin; I, intermediate resistance; B, bipolar; M, monopolar; St, subterminal; Pt, peritrichous; U, unknown.^11-23^

| Characteristic | 1 | 2 | 3 | 4 | 5 | 6 | 7 | 8 | 9 | 10 | 11 | 12 | 13 | 14 | 15 | 16 | 17 | 18 | 19 | 20 | 21 | 22 | 23 | 24 | 25 | 26 | 27 | 28 | 29 | 30 |
| --- | --- | --- | --- | --- | --- | --- | --- | --- | --- | --- | --- | --- | --- | --- | --- | --- | --- | --- | --- | --- | --- | --- | --- | --- | --- | --- | --- | --- | --- | --- |
| Oxidase | + | + | + | + | + | + | + | + | + | + | + | + | + | + | + | + | + | + | + | + | + | + | + | + | + | + | + | + | + | + |
| Catalase | + | - | + | + | + | + | + | + | + | + | + | + | + | + | + | - | + | + | (+) | + | + | + | (+) | (-) | + | + | + | + | + | + |
| Nitrite reduction | - | - | - | (-) | - | - | + | - | + | - | + | + | + | - | ± | - | - | + | + | + | + | + | - | + | + | + | + | - | - | - |
| Indoxyl acetate hydrolysis | + | (-) | ± | - | - | - | - | + | - | + | - | - | + | + | + | + | - | - | (-) | - | - | + | + | - | - | + | - | + | - | + |
| Urease | - | - | - | - | + | + | + | + | - | + | + | + | + | - | - | - | + | - | - | - | - | + | - | - | + | + | - | + | - | - |
| Phosphatase activity | + | + | + | + | ± | + | + | - | + | - | + | U | + | - | - | + | - | + | (-) | + | + | + | ± | - | - | U | + | - | - | - |
| γ-Glutamyl transferase | - | - | - | + | + | U | U | - | - | + | + | U | + | - | - | U | + | - | U | - | - | U | U | U | + | U | + | - | - | - |
| Growth at 42°C | + | + | + | - | - | (-) | - | + | + | + | - | ± | ± | + | + | + | + | + | ± | - | - | ± | (-) | - | - | - | + | + | + | + |
| 1% glycine | - | - | - | - | - | - | U | + | - | - | - | + | - | + | - | - | U | + | - | + | - | - | - | - | - | + | - | + | - | + |
| Resistance to NA (30 mg) | + | + | + | + | - | + | U | - | - | - | I | + | + | - | ± | - | ± | I | - | I | + | - | - | - | U | + | - | - | + | + |
| Resistance to CE (30 mg) | - | + | + | + | + | - | U | + | + | + | + | + | - | + | + | (-) | + | + | + | + | + | - | - | + | U | + | + | + | + | + |
| Periplasmic fibers | - | - | - | - | + | - | - | - | - | + | + | + | - | - | - | - | - | - | - | + | - | + | - | - | - | - | - | + | - | - |
| Distribution of flagella | M | B | B | B | B | M | B | St | B | B | B | B | B | St | B | B | B | M | B | B | M | B | B | B | B | B | B | B | M | B |
| Number of flagella | 1 | 2 | 2 | 7-14 | 6-12 | 2-5 | 6-8 | 2 | 2 | 7-10 | 11-22 | 3-4 | 10-20 | 2 | 2 | 2 | 2 | 2 | 1-2 | 6-12 | 1 | 14-20 | 2 | 2 | 4-10 | 2 | 1-2 | 7-14 | 1 | 2 |
| DNA G+C content (mol%) | 41 | 40 | 39 | 35 | 32 | 30 | 37 | 30 | 40 | 36 | 45 | 35 | 46 | 39 | 34 | 48 | 36 | 35 | 37-38 | 44 | 38 | 45 | 35 | 37 | 47 | 36 | 40 | 41 | 38 | 41 |

| Characteristic | 31 | 32 | 33 | 34 | 35 | 36 | 37 | 38 | 39 | 40 | 41 | 42 | 43 | 44 | 45 | 46 | 47 | 48 | 49 | 50 | 51 | 52 | 53 | 54 | 55 | 56 | 57 | 58 | 59 | 60 |
| --- | --- | --- | --- | --- | --- | --- | --- | --- | --- | --- | --- | --- | --- | --- | --- | --- | --- | --- | --- | --- | --- | --- | --- | --- | --- | --- | --- | --- | --- | --- |
| Oxidase | + | U | + | + | + | + | + | + | + | + | + | + | + | + | + | + | + | + | + | + | + | + | + | + | + | + | + | + | + | + |
| Catalase | + | + | + | + | + | (+) | + | + | + | + | + | + | + | + | - | + | + | + | + | + | + | + | + | + | + | + | + | - | - | + |
| Nitrate reduction | - | + | - | + | + | + | - | + | - | - | - | + | + | - | - | + | + | - | + | - | + | + | + | - | + | + | - | - | - | - |
| Indoxyl acetate hydrolysis | - | U | - | + | - | - | (-) | - | - | (-) | - | U | - | + | - | - | - | - | + | - | + | - | - | - | - | - | - | + | + | - |
| Urease | + | - | + | + | - | - | (+) | - | - | + | + | + | - | ± | - | + | + | + | + | - | + | - | - | - | + | + | + | - | - | + |
| Phosphatase activity | + | + | - | + | + | - | + | - | - | ± | + | (-) | - | - | ± | + | + | + | + | + | + | + | + | + | + | + | - | + | + | - |
| γ-Glutamyl transferase | - | - | U | U | U | U | + | - | + | + | + | + | - | - | + | + | + | + | - | - | + | U | + | + | + | + | + | - | + | + |
| Growth at 42°C | + | + | - | ± | + | + | (-) | + | + | - | - | + | + | + | - | - | - | - | + | + | - | + | + | + | - | - | - | + | + | - |
| 1% glycine | + | - | - | - | + | - | - | + | + | - | - | - | + | + | - | - | - | - | + | + | + | - | - | + | - | - | - | - | U | - |
| Resistance to NA (30 mg) | + | - | - | - | - | + | (+) | + | + | - | U | + | - | ± | + | U | U | U | - | + | - | + | - | U | - | - | - | + | U | - |
| Resistance to CE (30 mg) | + | + | - | + | + | - | (-) | + | + | ± | U | + | + | + | + | U | U | U | + | + | + | + | + | U | - | - | - | + | U | - |
| Periplasmic fibers | - | - | + | - | - | - | - | - | + | - | - | + | - | - | - | - | - | - | - | - | - | U | - | - | - | - | + | - | U | - |
| Distribution of flagella | B | B | B | Pt | B | M | M | B | B | B | B | B | B | M | M | B | B | B | St | St | M | B | B | B | B | B | B | B | B | B |
| Number of flagella | 2 | 2 | 10-14 | 4-8 | 2 | 1 | 4-8 | 2 | 6-12 | 10-23 | 4-10 | 5-7 | 1-2 | 1 | 1 | 6–14 | 6–8 | 2–17 | 2 | 2 | 1 | 2 | 2 | 2 | 5-10 | 8-10 | 5-10 | 2 | 2 | 2-6 |
| DNA G+C content (mol%) | 40 | 34 | 34 | 43 | 38 | 34-35 | 35-37 | 37 | 35 | 46 | 40 | 33 | 39 | 32 | 33 | 48 | 47 | 47 | 32 | 38 | 34 | 34 | 32 | 38 | 47 | 46 | 48 | 35 | 45 | U |

References

1. Ho SA, Hoyle JA, Lewis FA, Secker AD, Cross D, Mapstone NP, et al. Direct polymerase chain reaction test for detection of Helicobacter pylori in humans and animals. J Clin Microbiol. 1991;29(11):2543-9.

2. Hazell SL, Eichberg JW, Lee DR, Alpert L, Evans DG, Evans DJ, Jr., Graham DY. Selection of the chimpanzee over the baboon as a model for Helicobacter pylori infection. Gastroenterology. 1992;103(3):848-54.

3. Turbett GR, Hoj PB, Horne R, Mee BJ. Purification and characterization of the urease enzymes of Helicobacter species from humans and animals. Infect Immun. 1992;60(12):5259-66.

4. Mackie JT, O'Rourke JL. Gastritis associated with Helicobacter-like organisms in baboons. Vet Pathol. 2003;40(5):563-6.

5. Arora S, Nedrud J, Czinn S. A novel primate model for Helicobacter pylori pathogenesis and vaccine development. Journal of Pediatric Gastroenterology & Nutrition. 2005;41(4).

6. Curry A, Jones DM, Eldridge J. Spiral organisms in the baboon stomach. Lancet. 1987;2(8559):634-5.

7. Curry A, Jones DM, Skelton-Stroud P. Novel Ultrastrnctural Findings in a Helical Bacterium Found in the Baboon (Papio anubis) Stomach. Journal of General Microbiology. 1989;135(8).

8. Pu Y, Zeng D, Yan H-j, Qu Y, Li Y-f, Li Y, et al. Gastrointestinal microbiota of a clinically dead hamadryas baboon ( Papio hamadryas ) by high-throughput sequencing. Chinese Journal of Microecology. 2022;34(10):1139-45.

9. Garcia A, Xu S, Dewhirst FE, Nambiar PR, Fox JG. Enterohepatic Helicobacter species isolated from the ileum, liver and colon of a baboon with pancreatic islet amyloidosis. J Med Microbiol. 2006;55(Pt 11):1591-5.

10. Al-Soud WA, Bennedsen M, On SLW, Ouis IS, Vandamme P, Nilsson HO, et al. Assessment of PCR-DGGE for the identification of diverse Helicobacter species, and application to faecal samples from zoo animals to determine Helicobacter prevalence. J Med Microbiol. 2003;52(Pt 9):765-71.

11. Zanoni RG, Piva S, Florio D, Bassi P, Mion D, Cnockaert M, et al. Helicobacter apri sp. nov., isolated from wild boars. Int J Syst Evol Microbiol. 2016;66(8):2876-82.

12. Collado L, Jara R, Gonzalez S. Description of Helicobacter valdiviensis sp. nov., an Epsilonproteobacteria isolated from wild bird faecal samples. Int J Syst Evol Microbiol. 2014;64(Pt 6):1913-9.

13. On SLW, Miller WG, Houf K, Fox JG, Vandamme P. Minimal standards for describing new species belonging to the families Campylobacteraceae and Helicobacteraceae: Campylobacter, Arcobacter, Helicobacter and Wolinella spp. Int J Syst Evol Microbiol. 2017;67(12):5296-311.

14. Aydin F, Saticioglu IB, Ay H, Kayman T, Karakaya E, Abay S. Description of the two novel species of the genus Helicobacter: Helicobacter anatolicus sp. nov., and Helicobacter kayseriensis sp. nov., isolated from feces of urban wild birds. Syst Appl Microbiol. 2022;45(4):126326.

15. Aydin F, Tarhane S, Karakaya E, Abay S, Kayman T, Guran O, et al. Helicobacter cappadocius sp. nov., from lizards: The first psychrotrophic Helicobacter species. Syst Appl Microbiol. 2024;47(6):126557.

16. Chan N, Shen Z, Mannion A, Kurnick S, PopescuIoana IS, Burton FJ, et al. Helicobacter cyclurae sp. Nov., Isolated From Endangered Blue Iguanas (Cyclura lewisi). Frontiers in Ecology and Evolution. 2021;9.

17. Gruntar I, Kostanjsek R, Pirs T, Papic B. Helicobacter colisuis sp. nov., isolated from caecal contents of domestic pigs (Sus scrofa domesticus). Int J Syst Evol Microbiol. 2022;72(11).

18. Gruntar I, Papic B, Pate M, Zajc U, Ocepek M, Kusar D. Helicobacter labacensis sp. nov., Helicobacter mehlei sp. nov., and Helicobacter vulpis sp. nov., isolated from gastric mucosa of red foxes (Vulpes vulpes). Int J Syst Evol Microbiol. 2020;70(4):2395-404.

19. Kawamura Y, Fujimoto Y, Kutsuna R, Tomida J, Yamamoto KI, Miyoshi-Akiyama T, et al. Helicobacter kumamotonensis sp. nov., isolated from human clinical specimens. Int J Syst Evol Microbiol. 2023;73(3).

20. Lopez-Cantillo M, Vidal-Veuthey B, Mella A, de la Haba RR, Collado L. Helicobacter ibis sp. nov., isolated from faecal droppings of black-faced ibis (Theristicus melanopis). Int J Syst Evol Microbiol. 2023;73(11).

21. Rimbara E, Aoki S, Suzuki M, Kobayashi H, Nakagawa T, Goto-Koshino Y, et al. Characterization of three novel Helicobacter species infecting stomachs of dogs and cats: Helicobacter gastrocanis sp. nov., Helicobacter gastrofelis sp. nov., and Helicobacter felistomachi sp. nov. Front Microbiol. 2024;15:1459401.

22. Shen Z, Mannion A, Lin M, Esmail M, Bakthavatchalu V, Yang S, et al. Helicobacter monodelphidis sp. nov. and Helicobacter didelphidarum sp. nov., isolated from grey short-tailed opossums (Monodelphis domestica) with endemic cloacal prolapses. Int J Syst Evol Microbiol. 2020;70(12):6032-43.

23. Wang H, Gu Y, Zhou G, Chen X, Zhang X, Shao Z, Zhang M. Helicobacter zhangjianzhongii sp. nov., isolated from dog feces. Front Genet. 2023;14:1240581.
